# On Retro-Uterine Hematocle and Non-encysted Sanguineous Effusions of the Peritoneal Cavity of the Pelvis, Considered as Accidents of Menstruation

**Published:** 1860-09

**Authors:** 


ART. V.—*De l'Hématocèle Rétro-Utérine et des Épanchements Sanguins non Enkystés de la Cavité Péritonéale du Petit Bassin, considérés comme des Accidents de la Menstruation.* Par AUGUSTE VOISIN.

*On Retro-Uterine Hematocele and Non-encysted Sanguineous Effusions of the Peritoneal Cavity of the Pelvis, considered as Accidents of Menstruation.* By AUGUSTUS VOISIN, M.D., etc. With a Plate. 8vo., pp. 468. Paris: J. B. Baillière and Son, 1860.

LOOKING at the large size of this book, and the infrequency of the disease of which it treats, we need hardly say that its author has completely exhausted his subject, having drained it to the very dregs. He has left no stone unturned in pursuit of his facts, but has thoroughly explored every nook and corner of the literature of the profession likely to throw any light upon retro-uterine hematocele and bloody effusions into the pelvic cavity. His first inquiries were embodied in his Inaugural Dissertation, which was subsequently elaborated, step by step as it were, into the present monograph, a pretty octavo of nearly five hundred pages. As books of reference, such productions are invaluable, especially to lecturers and writers, affording as they do a complete history of the respective subjects which they discuss. We are therefore greatly indebted to the talented and accomplished young author, M. Voisin, for the able and faithful manner in which he has discharged his duty in the work of which we are about to give a brief notice. It is doubtless only the precursor of other and more valuable treatises.

M. Voisin divides the historical portion of his work into two parts: the first comprehending all the cases of retro-uterine hematocele which occurred prior to 1850, and which, scattered through the different medical journals, were published by their respective observers without any idea of the real nature of the disease they had described; and the second, commencing at that period when Professor Nélaton gave to this affection a definite position in nosology. Passing by this portion of the monograph as possessing more of a literary than a practical interest, we plunge at once into *medias res*, giving a close analysis of such parts as shall tend to place the contents of the work fairly before the reader.

The term retro-uterine hematocele is employed to designate an encysted tumor containing blood, situated in the peritoneal cavity of the pelvis, between the uterus and the rectum, the invariable result of accidents of menstruation. The disease is by no means frequent, there being but fifty cases on record. Menstrual disorder may also give rise to a hemorrhage into the same situation; but the loss of blood is so rapid that time is not afforded for its becoming encysted, and the patient consequently dies either from prostration or from peritonitis. The circumstances which predispose to the formation of retro-uterine hematocele relate to the condition of the patient at the time of its occurrence. The age is mentioned in thirty-four cases, the average being thirty years, at which period the venereal passion is most strong. The temperament of ten subjects was nervous, of four sanguine, and of one hysterical. The alteration of the blood which takes place during eruptive fevers seems to predispose to pelvic hemorrhages, and in a majority of the cases the menstrual flux was abundant, and of long duration, the blood was frequently clotted, and the act of menstruation was painful. In twenty-five instances, the time of the first appearance of menstruation was noted, the average age being between fourteen and sixteen years. Constipation, great sexual indulgence during or soon after menstruation, and blows upon the pelvis, may also give rise to the affection.

The immediate *causes* of retro-uterine hematocele may be classed under three heads, namely: congestion and hemorrhage from the ovarian vesicles arising during menstruation; reflux of blood from the uterus into the Fallopian tubes and the peritoneal cavity; hemorrhage from the Fallopian tube, the blood issuing from the mucous membranes of the oviduct and uterus, and from the proper tunic of the Graffian vesicle during the act of menstruation. The first cause is by far the most frequent, and three distinct pathological conditions may produce it, namely: venous congestion of the ovary and of its capillaries; a condition of the blood characterized by an increase or diminution of its fibrin; and, lastly, the hemorrhagic diathesis. Tubal hemorrhage is rare, but is favored by the physiological sanguineous flow, of which the tube is the seat during menstruation, and at least two cases may be traced to this cause. But one case can be assigned to reflux of blood from the uterus into the Fallopian tube and into the cavity of the peritoneum, and for its production the body of the uterus must be flexed upon its neck, thus affording an obstacle to the blood flowing into the vagina. Being thus retained, the cavity of the uterus becomes dilated, and the blood being unable to escape at the os, passes into one or both of the oviducts.

Non-encysted bloody effusions may arise from the same causes as retro-uterine hematocele, but, unlike the latter, may be produced by a varicose condition and rupture of the veins on the inferior surface of the ovary. In this case large vessels being involved, the loss of blood is great, and produces death from exhaustion or from peritonitis.

The relative frequency of the points of origin of the blood in cases of retro-uterine hematocele is difficult to be determined, on account of the resulting inflammation; but in the instances in which it was discovered, it was found that it issued from the ovary six times, and twice from the Fallopian tubes. In the cases of non-encysted effusions, the seat of the bleeding was in six cases the ovary, in three the uterine cavity, in four the Fallopian tubes, and in four ovarian varix.

The *symptoms* of non-encysted sanguineous effusions into the pelvis come on suddenly in the midst of good health, or coincident with menstruation. The patient is seized with excruciating pains in the lower portion of the abdomen, the pulse is small and almost imperceptible, the skin is cold and blanched, the belly is hard and tense, hiccough and vomiting supervene, and finally syncope or complete collapse sets in, followed by death, in all cases, in less than twelve hours, from peritonitis.

The symptoms of retro-uterine hematocele may come on suddenly or gradually, the patient soon complaining of great pain, aggravated by the slightest pressure or movement, and nearly always limited to the pelvic cavity. It increases at every menstrual period, and is sometimes complicated with a very fatiguing sensation of weight at the anus. A few days having elapsed, a tumor may be detected above the pubes, usually larger on the right side of the hypogastrium than on the left, at first soft and fluctuating, and gradually becoming hard and irregular. It may be felt through the vagina pushing forward the neck of the uterus, and flattening the rectum behind, stretching forcibly the walls of the vagina, and very often occupying the inferior strait, being about twenty lines distant from the vulvar orifice. The neck of the uterus is carried forcibly forward against the posterior surface of the pubic symphysis, and micturition and defecation are very difficult. The constitutional symptoms are those of peritonitis, and consist of nausea, vomiting, intense fever, abdominal pains, the Hippocratic countenance, and a blanched appearance of the surface. From the very moment of its formation, the tendency of the tumor is to diminish; but nature requires some time to effect the absorption of the blood, and in some cases the fibrin not being taken up, becomes mixed with the serum poured out by the inflamed peritoneum, and the result is a black, viscous fluid resembling molasses. The morbid condition of the mixture excites inflammation of the retro-uterine pouch, and may provoke ulceration of one of its walls, and thus lead to the spontaneous evacuation of its contents.

The *duration* of retro-uterine hematocele is very variable. The period of the disappearance of the tumor in ten cases of cure was noticed nine times, being in one six weeks, in three three months, in two four months, in two six months, and in one eight months. Of ten cases of death, in all but one was the period of this termination reported; from which we find that three cases died in three months, one in four months, one in eight months, one in fifteen days, one in ten days, one in seven days, and one in four days. In comparison with a cure, death is very infrequent, and when the case is not interfered with, a cure is the rule, while operations practiced for the evacuation of the fluid have frequently led to death, or excited symptoms of purulent infection.

When the case is left to nature for a cure it may terminate in several ways—1st, by the absorption of the tumor; 2dly, by evacuation of the liquid into the rectum; 3dly, by opening into the vagina; 4thly, by the effusion of the fluid into some portion of the healthy peritoneal cavity; and, 5thly, by suppuration of the contents of the tumor. The first termination took place fifteen times out of twenty-five cases not subjected to surgical interference, and in seven cases is the time of this occurrence mentioned, being in two after one month and a half, in three after four months, in one after six, and in one after eight months. The absorption takes place only during the menstrual period, and repose in bed is absolutely necessary for its proper performance. As the tumor diminishes, the fever abates, the pains lose their acute character, defecation and micturition become more easy, appetite returns, and the general health becomes greatly improved. Evacuation of the contents of the tumor by the rectum took place six times out of twenty-seven cases in which the mode of termination is noted. In all these high constitutional disturbance existed, and two of the patients died. Evacuation of the fluid through the vagina occurred in three instances, and in all with a happy result. Effusion of the blood into the peritoneal cavity took place in four cases, and all died of peritonitis. Suppuration of the contents of the sac is very rare, and requires surgical interference, being characterized by the ordinary local and general symptoms of this morbid action.

The *diagnosis* of non-encysted effusions of blood into the pelvis is somewhat difficult, as a few of the symptoms resemble those of poisoning; and although there are evident signs of an internal hemorrhage, yet it is almost impossible to say from what organ the blood proceeds. The accident may, however, in the generality of cases, be known by its rapid course and coincidence with menstruation; atrocious abdominal pains; a small, soft pulse; hiccough; vomiting; swelling of the belly; coldness of the surface; rapid blanching of the skin, and syncope.

The diagnosis of retro-uterine hematocele rests upon the abundance of the menses; the coincidence of the appearance of the affection and menstruation, which is most often abnormal; the rapid progress of the symptoms, which resemble those of peritonitis; the large and quick development of the uterus, and the tilting forward of its neck; the presence of a tumor in the utero-rectal cul-de-sac; and the character of the pains, which the patient compares to those of labor.

The author enters very fully into the differential diagnosis between retro-uterine hematocele and peri- and retro-uterine abscess, ovaritis, ovarian cysts, encephaloid, extra-uterine fœtation, fibrous tumors of the uterus, retroversion and retroflexion of the organ, varix of the broad ligaments, fæcal accumulations, and other affections; and then discusses the question as to the tumor being intra-peritoneal, extra-peritoneal, or both, and arrives at the conclusion that it is always contained in the peritoneal cavity, no well-authenticated case having been reported to disprove this position. As our limits are nearly exhausted, we must refer the reader to the work itself for many interesting details, which our short space prevents us placing before him.

The *treatment* is divided into prophylactic and curative, but in regard to the former it is almost impossible to put it into practice, as it is difficult to foresee that a woman may become the subject of an intra-pelvic hemorrhage, or of a retro-uterine hematocele. The conditions favorable to the production of one or the other of these affections are a passionate, sanguineous temperament; painful, irregular, and very copious menstruation; and a varicose condition of the veins of the inferior extremities, the anus, or the vagina. Moreover, as these accidents have been produced by excessive coitus, particularly at the time of menstruation, we should advise abstainment from this during the catamenial period, and for some days after the flow has ceased. The curative treatment of non-encysted sanguineous effusions consists in endeavoring to arrest the loss of blood, and for this, besides other measures, small bleedings of six or seven ounces may be resorted to. The remainder of the treatment resolves itself into the application of cold to the belly, sinapisms to the upper extremities, absolute rest, and the avoidance of all excitement.

The curative treatment of retro-uterine hematocele may be either surgical or medical, the former consisting in evacuating the contents of the sac through the vagina, either by puncture with a trocar or by incision, and afterwards injecting, several times a day, tepid water. Or, after an opening has been made, a gum-elastic tube may be introduced, which favors a constant discharge, and through which injections may readily be made. In any case, the patient must be confined to her bed until a cure has been effected. The medical treatment consists in endeavoring to prevent new hemorrhages by enjoining absolute rest, keeping the pelvis well elevated, the application of cold compresses to the hypogastrium, and of revulsives to the upper extremities, and the internal administration of acids and astringents. To promote an absorption of the tumor, cataplasms may be applied to the belly, and after the cessation of acute symptoms, purgatives may be administered, and flying blisters be applied to the hypogastrium. The anæmic condition of the patient may be improved by the exhibition of iron, tonics, and a generous diet, and peritonitis is to be combated by venesection, calomel, opium, and vesicants.

The medical treatment contrasts most favorably with the surgical. Thus of twenty-seven cases subjected to the former only five died, whereas of twenty cases in which the sac was evacuated but fifteen recovered. The remainder of the work, comprising one hundred and twelve pages, is taken up with the details of thirty-six cases of this affection.

